# Climate change is predicted to alter the current pest status of *Globodera pallida* and *G. rostochiensis* in the United Kingdom

**DOI:** 10.1111/gcb.13676

**Published:** 2017-03-30

**Authors:** Laura M. Jones, Ann‐Kristin Koehler, Mirek Trnka, Jan Balek, Andrew J. Challinor, Howard J. Atkinson, Peter E. Urwin

**Affiliations:** ^1^ School of Biology University of Leeds Leeds UK; ^2^ Institute of Climate and Atmospheric Science School of Earth and Environment University of Leeds Leeds UK; ^3^ Global Change Research Centre Czech Academy of Sciences Brno Czech Republic; ^4^ Department of Agrosystems and Bioclimatology Mendel University Brno Brno Czech Republic; ^5^ Department of Climate Change Impacts on Agroecosystems Global Change Research Institute CAS CZECHGLOBE Brno Czech Republic

**Keywords:** climate change, *Globodera pallida*, *Globodera rostochiensis*, plant pathogens, potato cyst nematode, soil temperature simulations, soil‐borne pests

## Abstract

The potato cyst nematodes *Globodera pallida* and *G. rostochiensis* are economically important plant pathogens causing losses to UK potato harvests estimated at £50 m/ year. Implications of climate change on their future pest status have not been fully considered. Here, we report growth of female *G. pallida* and *G. rostochiensis* over the range 15 to 25°C. Females per plant and their fecundity declined progressively with temperatures above 17.5°C for *G. pallida*, whilst females per plant were optimal between 17.5 and 22.5°C for *G. rostochiensis*. Relative reproductive success with temperature was confirmed on two potato cultivars infected with either species at 15, 22.5 and 25°C. The reduced reproductive success of *G. pallida* at 22.5°C relative to 15°C was also recorded for a further seven host cultivars studied. The differences in optimal temperatures for reproductive success may relate to known differences in the altitude of their regions of origin in the Andes. Exposure of *G. pallida* to a diurnal temperature stress for one week during female growth significantly suppressed subsequent growth for one week at 17.5°C but had no effect on *G. rostochiensis*. However, after two weeks of recovery, female size was not significantly different from that for the control treatment. Future soil temperatures were simulated for medium‐ and high‐emission scenarios and combined with nematode growth data to project future implications of climate change for the two species. Increased soil temperatures associated with climate change may reduce the pest status of *G. pallida* but benefit *G. rostochiensis* especially in the southern United Kingdom. We conclude that plant breeders may be able to exploit the thermal limits of *G. pallida* by developing potato cultivars able to grow under future warm summer conditions. Existing widely deployed resistance to *G. rostochiensis* is an important characteristic to retain for new potato cultivars.

## 1 Introduction

Climate change has the potential to alter the distribution of animals, but outcomes can vary. In the absence of habitat management, it may result in future extinction of some species, as reported for drought‐sensitive butterflies in the United Kingdom (Oliver et al., 2015). Worldwide, both vertebrate and invertebrate species have moved towards higher latitudes over a circa 25‐year period (Chen, Hill, Ohlemuller, Roy, & Thomas, 2011; Hickling, Roy, Hill, Fox, & Thomas, 2006). Data for many of the 612 crop pests and pathogens analysed established a global move poleward since the 1960s for some organisms but not for either *Globodera pallida* or *G. rostochiensis* in the Northern Hemisphere (Bebber, Ramotowski, & Gurr, 2013). Both these species of potato cyst nematodes (PCN) occur throughout the potato‐growing regions of the United Kingdom (Minnis et al., 2002) and have been reported throughout Europe, Latin America and parts of Asia, North America, Oceania and Africa where potatoes are cropped (http://www.cabi.org/isc/datasheet/27033#20127201272). They cause losses to potato harvests estimated at £50 m/year in the United Kingdom alone and several times that value across Europe (http://www.cabi.org/isc/datasheet/27033).

Current management relies on nematicides, resistant potato cultivars and long‐crop rotations. However, several nematicides have recently been banned under current EU legislation (Regulation (EC) No 1107/2009). There are few cultivars grown widely with high levels of resistance particularly to *G. pallida*. Early success in breeding resistance to *G. rostochiensis* was achieved with a single gene from *Solanum tuberosum* ssp. *andigena*. It has provided durable, qualitative resistance to this nematode in cultivars such as Maris Piper. Breeding for resistance to the more common forms of *G. pallida* is more complex. No single gene offers complete resistance to all populations of this nematode which vary in the level of virulence they offer to partially resistant cultivars (Dalton, Griffin, Gallagher, de Vetten, & Milbourne, 2013). Rotational control is an important pest management strategy for PCN that counters their reproductive success on a host plant by allowing natural decline rates when other crops are grown (http://potatoes.ahdb.org.uk/online-toolbox/pcn-calculator).

PCN are host‐specific parasites that co‐evolved over 15–21 × 10^6^ years with wild potato species (Solanum L. section Petota Dumort.) of which 130 species are recognized in Peru and Bolivia (Spooner & Hijmans, 2001). The climate of the Andean highlands pre‐adapted both the two PCN species and their host potato plants to cool‐temperate climates worldwide where the crop is now grown. *G. pallida* is adapted to high altitudes and is considered to have undergone an expansion northwards within Peru in the Miocene as the Andean chain rose in that region. Phylogenetic analysis of *G. pallida* populations has been used as a molecular clock to determine when an altitude threshold of 2.0–2.5 km was reached for the elevating Andes in different regions of Peru. *G. rostochiensis* is assumed to originate from where uplift of the paleo Andes was less extreme, and therefore, the climate is slightly warmer (Plantard et al., 2008).

The females of both *Globodera* species retain all eggs within a cyst formed by tanning of their body walls at death. There is normally a single generation per potato crop, and the encysted eggs remain dormant until infective juveniles hatch from them in response to root diffusate from potato plants (Forrest & Farrer, 1983; Perry & Beane, 1982). A partial second generation has been observed for some populations of *G. rostochiensis* in the United Kingdom (Evans, 1969; Jones, 1950) and Italy (Greco, Brandonisio, Tirro, & de Marinis, 1988). Potatoes are grown widely in England and Scotland with planting of main crops from mid‐April and harvest up to early October (Daccache et al., 2012; Gregory & Marshall, 2012). More than half the national potato plantings occur in Eastern England and Yorkshire, about 22% in Scotland, about 12% in the West Midlands and the remaining 14% in other parts of England and Wales. Females develop after the juveniles invade potato roots. They emerge through the cortex onto the root surface and continue to grow on main crop potatoes in the United Kingdom from June onwards. For example, first emergence of females onto the root surface occurred in Southern England in late June, 55 days after planting in early May (Whitehead, 1992) and mid‐June for early planted potatoes in Belgium (Ebrahimi, Viaene, Demeulemeester, & Moens, 2014).

**Figure 1:**
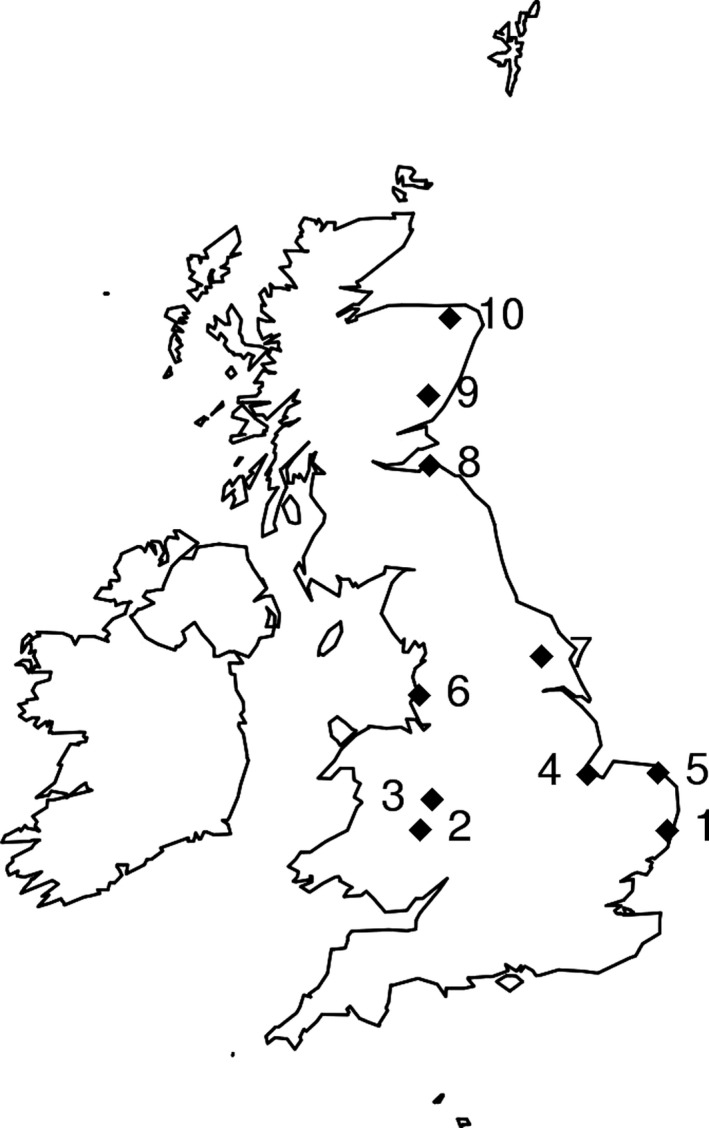
Locations of selected 5 × 5 km weather grid cells

The difference in the altitude adaptation of the two PCN species in the Andes, together with previous work, suggested a comparative approach for this study to define if their likely responses differ to the future increase in summer temperatures that have been projected for the United Kingdom (Jenkins et al., 2009; Parker, Legg, & Folland, 1992; Trenberth et al., 2007). Being soil borne, nematodes respond to soil rather than air temperature. As soil temperatures are rarely recorded and are not outputted from global climate models, modelling studies tend to use air instead of soil temperatures. García‐Suárez and Butler (2006) showed for three sites in Ireland that over the last century annual mean soil temperatures increased more than air temperature, and rises and falls do not occur at the same time. To represent recent and future soil temperatures for 10 sites across the UK potato‐growing area (Figure 1, Table S1), we validated a soil temperature model (SoilClim) (Hlavinka et al., 2011) for UK conditions. The soil temperature model was used together with recent and future climate data from a weather generator for medium‐ and high‐emission scenarios and three future time periods (http://ukclimateprojections-ui.metoffice.gov.uk/ui/admin/login.php). Female reproductive success of PCN at 15 to 25°C was determined and related to predicted future soil temperatures during time of female development for 10 sites covering the main potato‐growing areas of the United Kingdom. This enabled changes in pest status of PCN on UK potato crops in relation to climate change to be estimated.

## 2 Materials and Methods

### 2.1 The effect of temperature on growth of female *Globodera*

Tubers of *Solanum tuberosum* L. var. Desiree were grown in pots with a diameter of 18 cm containing sandy loam (1:1 loam soil:sand) in a glasshouse set at 20°C with a 16‐hr day length. Aliquots of 1,500 hatched, infective juveniles (J2s) of each species were added to the soil after three weeks of plant growth. Juveniles were hatched from *G. pallida* (pathotype Pa 2/3) or *G. rostochiensis* (pathotype Ro1) eggs within cysts at 20°C using root diffusate collected from three‐week‐old potato roots. J2s were washed four times in tap water and pipetted into the soil at three locations around the planted tuber at a density of one juvenile per μL water. Immediately after soil infestation, the potato plants were transferred to heat mats set at 15, 17.5, 20, 22.5 and 25°C in a glasshouse with a 16‐hr day length.

At least three replicate plants per temperature were grown for each time point. Soil temperature in each pot was monitored using an iButton (Maxim Integrated, San Jose, California, USA) and was within ±1°C of the set mat temperature throughout the experiment. Females were collected from roots at weekly intervals, from their first appearance on the root surface at three weeks until nine weeks postinfection by washing them through a series of 1000‐, 150‐ and 63‐μm sieves. Images of females were taken using a Leica MZ16 stereo‐binocular microscope and a MicroPublisher 3.3 RTV colour camera (QImaging, Surrey, BC, Canada). Projected surface area was measured in mm^2^ using Image‐Pro Analyzer 7.0 (Media Cybernetics Inc., Rockville, MD, USA). The eggs within some newly formed cysts of both species were counted after measuring their projected surface area to provide a calibration curve that relates area to egg number.

### 2.2 Population growth of *Globodera* on a range of cultivars at 15, 22.5 and 25°C

Tubers of nine potato cultivars widely grown in the United Kingdom (*Solanum tuberosum* L. var. Arsenal, Cara, Desiree, Estima, Innovator, Markies, Melody, Maris Peer and Maris Piper) were planted and grown as described above with three or four replicates per temperature for each potato cultivar in soil containing *G. pallida* (pathotype Pa 2/3) at a density of 5 eggs g^−1^. The plants were grown on heat mats set at 15, 22.5 (all cultivars) and 25°C (Desiree and Maris Peer only) in a glasshouse with a 16‐hr day length. After twelve weeks of growth, plants and soil were allowed to dry. Roots and soil were mixed together, and three 100 g samples were collected from each pot. Egg and cyst counts were carried out using standard procedures by an agricultural extension company (ADAS UK Ltd, Wolverhampton, UK). Cysts were recovered from dried soil samples using a Fenwick can, they were opened and the number of eggs was quantified on a counting slide (see Southey 1986 for details). The same experiment was performed for *G. rostochiensis* with potato cultivars Desiree and Maris Peer.

### 2.3 The effect of fluctuating diurnal heat stress on females of *Globodera*

*Solanum tuberosum* L. var. Desiree were grown in a Sanyo^TM^ MLR‐350H Plant Growth Chamber (Moriguchi, Osaka prefecture, Japan) at 17.5°C with 16‐hr day length for three weeks before J2 of *G. pallida* (pathotype Pa 2/3) or *G. rostochiensis* (pathotype Ro1) were added to the soil as above. After further four weeks, six plants infected with each species were moved to a plant growth chamber with a cycling diurnal temperature. The remaining three plants for each species continued at a constant temperature of 17.5°C. The cabinets with cycling temperatures increased to a maximum value of 32.5°C over 4–5 hr and held the maximum temperature for 3–4 hr before the temperature fell over 4–5 hr to 17.5°C which was maintained for the remaining 12–13 hr of the 24‐hr cycle. The 8‐hr dark period occurred, whilst the plants were at 17.5°C. After one week, all plants were returned to the cabinet set at a constant temperature of 17.5°C and females were collected from the roots at six and seven weeks post nematode addition.

### 2.4 Potential for a second generation of *G. rostochiensis*

Eight *Solanum tuberosum* L. var. Desiree plants were grown for two weeks before 1,500 J2 *G. rostochiensis*/plant were introduced to each as described above. The temperature was 21.2 ± 1°C throughout the experiment. Half the plants were selected at random and harvested after nine weeks. The remaining plants were harvested at 16 weeks. The number of cysts/100 g soil, the projected surface of each cyst and their egg content were measured as described earlier.

### 2.5 Dependency of development rate of *Globodera* on temperature

Temperature‐dependent development of *Globodera* females over time was investigated using the Gompertz model as modified by Zwietering, Jongenburger, Rombouts, and van't Riet (1990) to give the parameters a biological meaning:

(1) $$y\left(t\right)=A\exp\left\{-\exp\left[\frac{\mu_{m}\exp\left(1\right)}{A}\left(\lambda -1\right)+1\right]\right\}.$$

where μ_*m*_ is the maximum specific growth rate, that is the tangent in the inflection point; λ is the lag time before first egg production (*x*‐axis intercept of the tangent); and *A* is the asymptote which is defined as the maximal female surface area achieved. The lag time was set to a minimum of two weeks as production of embryonated eggs is not expected to occur before this time point.

### 2.6 Soil temperature simulations

Soil temperature at 10 and 20 cm depth was simulated using SoilClim (Hlavinka et al., 2011) which requires daily minimum and maximum air temperature, precipitation, radiation, latitude and altitude of the location as input data. All SoilClim simulations were performed for light and medium soils (Trnka et al., 2014) with notional planting and harvest dates in mid‐April and early October (Daccache et al., 2012; Gregory & Marshall, 2012). Soil temperature was simulated for all combinations of a constant (3 t ha^−1^) and variable canopy, with or without irrigation. For the variable canopy, the total amount of biomass cover was increased linearly from 0 to 18 t ha^−1^ during the initial crop development once it had emerged. It was maintained at 18 t ha^−1^ during mid‐season before a decrease from 18 to 10 t ha^−1^ in the late season. Irrigation was simulated by maintaining readily available water at a minimum of 40% until increased by rainfall (Daccache, Weatherhead, Stalham, & Knox, 2011). The length of the different potato plant developmental stages was set at 40 days for emergence, 60 days for crop development, 45 days for mid‐season and 28 days for late season (http://www.fao.org/nr/water/cropinfo_potato.html).

For the validation of SoilClim, two weather stations (Rothamsted and East Malling) from the Met Office Integrated Data Archive System (MIDAS) Land and Marine Surface Station Data were selected that provided hourly soil temperature at a depth of 10 cm and daily values for minimum and maximum air temperature and precipitation. Daily soil temperature data were computed from the hourly observations to compare to the SoilClim output. Using hourly observed soil temperature for comparison was necessary as daily soil temperature data at the MIDAS weather stations are recorded at 9 am and therefore do not correspond to daily average values. Solar radiation was not available and was therefore calculated from minimum and maximum air temperature (Hargreaves, Hargreaves, & Riley, 1985; Trnka, Zalud, Eitzinger, & Dubrovský, 2005) with Hargreaves constants A_h_ and B_h_ derived from European Commission (http://www.treemail.nl/download/treebook7/radiation/index.htm). No suitable data set could be found for soil temperatures at a depth of 20 cm. For the simulations, a light and medium soil type in combination with a constant canopy was assumed as satellite images confirmed that both sites had a grass cover.

### 2.7 Recent and future climate projections for potato‐growing locations across the United Kingdom

We used minimum and maximum temperature and precipitation provided by a weather generator (WG) (UKCP09 climate projections downloaded from http://ukclimateprojections-ui.metoffice.gov.uk/ui/admin/login.php) to represent recent and future climatic conditions for ten selected sites (5 × 5 km grid cells) covering the main potato‐growing areas of the United Kingdom (Figure 1, Table S1; http://potatoes.ahdb.org.uk/sites/default/files/styles/image-node/public/content/main%20production%20area.JPG?itok=_qLoBOpF; Daccache et al., 2012). The WG provided recent values (1961 to 1990) and three future time periods, that is 2040s (representing the time period 2030 to 2059), 2060s (2050 to 2079) and 2080s (2070 to 2099). The model was run with scenarios for both medium (corresponding to IPCC SRES A1B) and high (IPCC SRES A1FI) emissions (IPCC, 2000). For each time period and combination of emissions, 100 perturbations of the WG were combined with 50 plausible years to include uncertainty.

### 2.8 Estimating the population growth of *Globodera* in response to temperature changes across the United Kingdom

Predictions of future *Globodera* pressure were calculated using our data for the effect of temperature on female development and population size, together with simulated soil temperature at 10 and 20 cm depth during female development. The potato root system is concentrated in the upper 30 cm of the soil layer (Asfary, Wild, & Harris, 1983), with PCN distribution proportional to root length density (Storey, 1982). Therefore, we used estimates of root length density with depth to weight the simulated soil temperature. Soil layers 0 to 15 cm and 15 to 30 cm were assumed to correspond to SoilClim soil temperature estimates at 10 and 20 cm soil depth and were weighted 0.45 and 0.55, respectively (Asfary et al., 1983). The SoilClim simulations were based on a variable canopy with irrigation to provide realistic combinations for conditions experienced by PCN.

### 2.9 Statistical analyses

All data were analysed using a standard statistical package (SPSS v20; IBM Corporation Armonk, NY, USA; http://www-01.ibm.com/software/analytics/spss). All means are given with the standard error of the mean (*SEM*). The skewness associated with some sample means was determined. Stem and leaf analysis was applied to identify outlier values when the skew was significant. Another analysis carried out was anova using the general linear model multivariate procedure and one‐way analysis with both a priori contrasts and post hoc comparisons of means using the Student‐Newman‐Keuls (SNK). Curve fits in regression analysis were selected using the Akaike information criterion (AIC, extractAIC function from r stats package see https://stat.ethz.ch/R-manual/R-devel/library/stats/html/extractAIC.html). This combines the goodness of fit of a model and its complexity. Nonlinear regression was used to fit Gompertz curves to changes in projected surface areas of females with time. r version 3.1.2 was used for the analysis of the soil temperature data (http://www.R-project.org).

## 3 Results

### 3.1 The effect of temperature on growth of female *Globodera*

The mean projected surface areas of 2,899 and 2,398 collected females of *G. rostochiensis* and *G. pallida* respectively were measured over 3–9 weeks after adding juveniles to soil (Fig. S1). Gompertz curves were fitted to the data set with a minimum lag of two weeks before the first females were present on the root surface (minimum value of *R* ^2 ^= 0.77 except for *G. rostochiensis* at 25°C where *R* ^2 ^= 0.52). *G. rostochiensis* fitted a longer lag phase at 15°C but reached the same final size as other temperatures by nine weeks (Fig. S1a). The asymptote for females of *G. pallida* provided a smaller final projected surface area at 25°C than other temperatures (Fig. S1b). There was no significant difference in the areas between eight and nine weeks for either species at each temperature (a priori contrasts, one‐way anova), indicating that growth had been completed for all temperatures by that time. The data for final size at eight and nine weeks were therefore combined.

**Figure 2:**
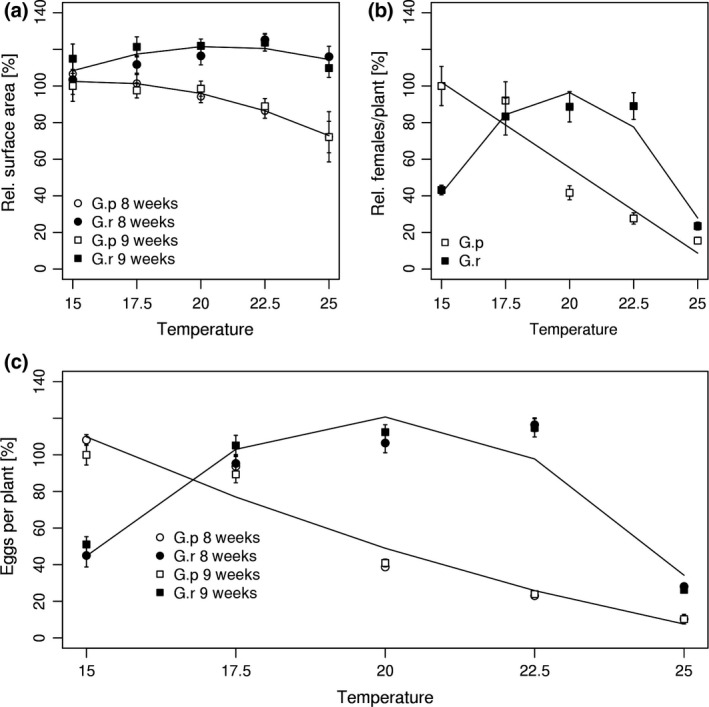
Final projected surface area (a) and maximum number of females of final size per plant (b) for the two species at eight and nine weeks combined. (c) The number of eggs produced based on accumulating the estimated egg content of each developed female from its projected surface area (see text for further detail). (a–c) are expressed as a percentage of the values at 15°C for *Globodera pallida* at nine weeks. All values are means ± *SEM*. Linear or quadratic curve fits were chosen according to the AIC criterion

The AIC method suggested the final projected surface area of female *G. rostochiensis* and *G. pallida* fitted a quadratic rather than a linear fit. The change in final projected surface area of its females over 15 to 25°C was much smaller for *G. rostochiensis* than for *G. pallida* that showed a clear reduction in projected surface area at higher temperatures (Figure 2a). Projected surface area at 25°C was reduced to 72 ± 10% of 0.165 ± 0.006 mm^2^ at 15°C (*p* < .01; a priori contrasts, one‐way anova). The number of females per plant of *G. rostochiensis* fitted a quadratic curve (*R* ^2 ^= 0.88) with optimal values of about 57 ± 6 at 17.5 to 22.5°C, whereas for *G. pallida*, the number decreased linearly (*p* < .01, *R* ^2 ^= 0.90) from 66 ± 7 at 15°C to 15 ± 2% at 25°C (Figure 2b). A linear relationship exists between the projected surface area of newly formed females of *G. pallida* and their egg content (Urwin, Atkinson, Waller, & McPherson, 1995). We found that there was no significant difference in this linear relationship between *G. pallida* and *G. rostochiensis* and therefore combined the data of the two species that resulted in the following equation: eggs/cyst = (1277.5 × area) – 37.1 with projected surface area in mm^2^. This calibration and number of females per plant enabled the number of eggs per plant at different temperatures to be estimated (Figure 2c).

**Figure 3:**
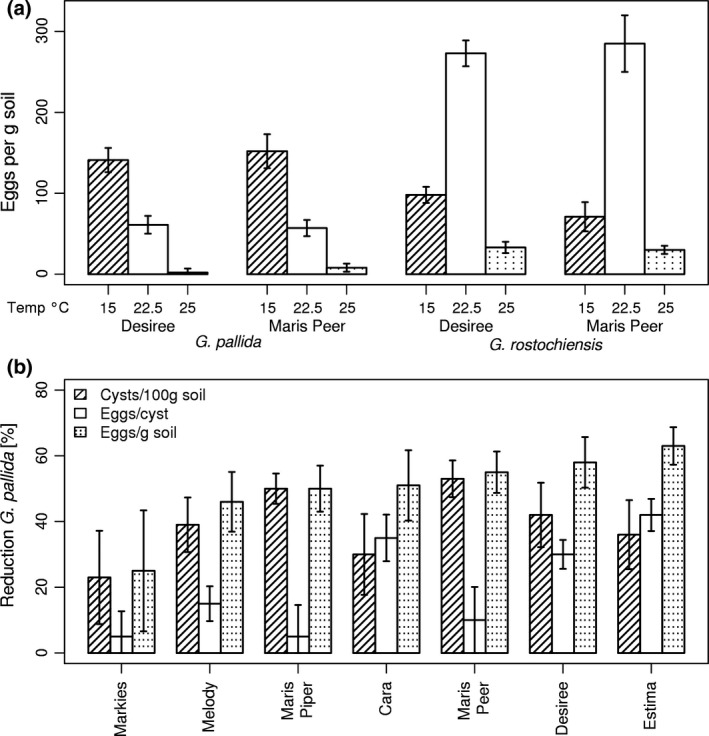
Eggs per g soil of *Globodera pallida* and *G. rostochiensis* at 15, 22.5 and 25°C for cultivars Desiree and Maris Peer (a) and reduction in cysts/100 g soil, eggs per female and eggs/g soil from multiplication of *G. pallida* on seven cultivars at 22.5°C relative to the corresponding values at 15°C (b). Values are means ± *SEM*

Reproductive success of *G. pallida* decreased with temperature from 15 to 25°C, whereas the optimum for *G. rostochiensis* was between 17.5 and 22.5°C. The results establish that *G. pallida* reproduces more successfully than *G. rostochiensis* at 15°C and is less productive at 20–22.5°C. Number of eggs/g soil produced by *G. pallida* and *G. rostochiensis* on cv Desiree and Maris Peer at 15, 22.5 and 25°C is given in Figure 3a (see Fig. S2 for number of cysts/100 g and egg content per cyst). For *G. pallida*, the results for the two cultivars were similar and so were combined. There was a highly significant reduction in eggs/g soil between both 15 and 22.5°C and also between the latter temperature and 25°C (*p* < .001, univariate anova, Figure 3a). Results for the two cultivars were also similar for *G. rostochiensis*. Both the decline from 22.5 to 25°C and the increase between 15 and 22.5°C were significant for Desiree and Maris Peer (at least *p* < .01, a priori contrast, one‐way anova, Figure 3a). Further analysis indicated that the effects for both species were mainly due to changes in cysts /100 g soil although eggs/ cyst were suppressed for both species on the two cultivars at 25°C (Fig. S2).

The comparative reproductive success of *G. pallida* at 15 and 22.5°C was studied for a further nine cultivars. The reduction in reproductive success for each cultivar is given in Figure 3b expressed as a percentage for each species at 22.5°C relative to their corresponding means at 15°C. Data collected for Arsenal and Innovator were excluded from the analysis due to low multiplication (<1) on these cultivars at both temperatures. The overall reduction from the mean for the remaining seven cultivars at the higher temperature was 39 ± 4% cysts/100 g soil, 21 ± 4% eggs/cyst and 50 ± 4% eggs/g soil. The reduction from means at 15°C was significant (<0.001 in all three comparisons, multivariate anova, pairwise comparisons, Bonferroni adjustment for multiple comparisons). There were no significant differences among cultivars for number of females or eggs/g soil (one‐way anova and SNK, *p* < .05, Figure 3b). The reduction in eggs/cyst for Estima at 22.5°C was significantly greater than for Maris Peer, Markies and Maris Piper (SNK, *p* < .05).

### 3.2 The effect of fluctuating diurnal heat stress on females of *Globodera*

Subjecting *Globodera* to a fluctuating diurnal heat stress from 17.5 to 32.5°C for seven days during 4–5 weeks after addition of J2 to the soil did not significantly affect their final number of eggs compared to the control grown at a constant 17.5°C. Females of *G. pallida* collected from plants subjected to the heat stress had a significantly smaller mean size of 0.133 ± 0.010 mm^2^ compared to the control of 0.176 ± 0.007 mm^2^ at one but not at two weeks after their return to 17.5°C (*p* < .05 and *p* = .29, respectively). No corresponding similar effect was detected for *G. rostochiensis* at either time point.

### 3.3 Potential for a second generation of *G. rostochiensis*

**Figure 4:**
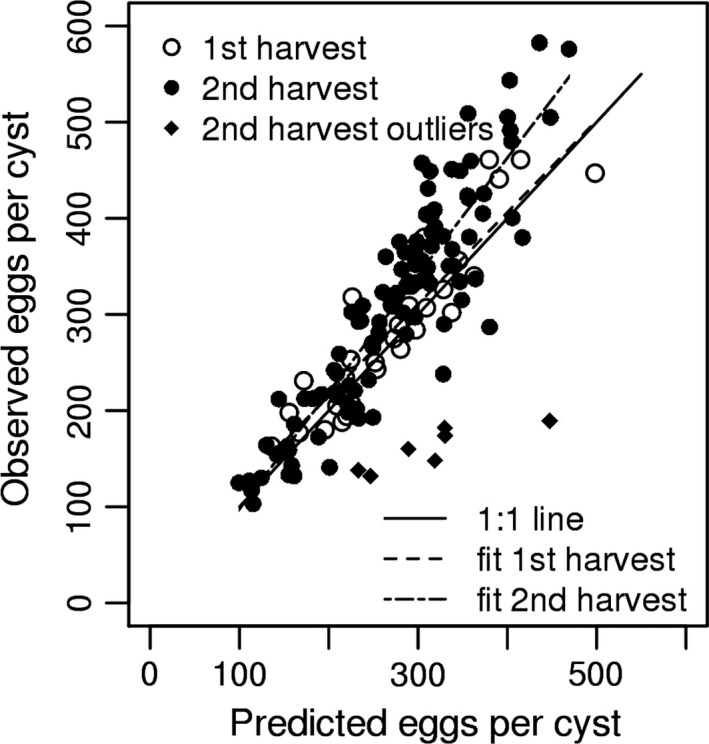
Number of observed eggs against that predicted from projected surface area for each cyst collected at nine weeks and 16 weeks from two batches of plants after infection of cv Desiree with hatched juveniles of *Globodera rostochiensis*. No outliers with low egg content for their size were detected at nine weeks, but seven outliers were present in the cysts recovered at 16 weeks

The number of cysts/ 100 g soil was 6.75 ± 1.17 (mean ± *SEM*) after nine weeks with a significantly higher value of 31.1 ± 6.58 after 16 weeks (*p* < .001; t test). Given that the number of cysts from the first generation of *G. rostochiensis* is complete by 7–8 weeks (Fig. S1a), the increase suggests a second generation contributed to the cyst collection at 16 weeks. The ratio of observed to predicted eggs had a mean for the first harvest of 1.071 ± 0.028 and 1.081 ± 0.020 for the second harvest. The distribution of values about the mean for the second, but not the first harvest, had a significant negative skew (−0.946 ± 0.226, *n* = 114, *p* < .01). Stem and leaf analysis suggested this was due to seven outliers with lower than expected observed egg content. Their exclusion eliminated the significant skew. These seven values are shown in Figure 4 but are not included in the regression line for the second harvest.

### 3.4 Recent and future climate projections for potato‐growing locations across the United Kingdom

SoilClim simulated soil temperatures at 10 cm soil depth were validated using observed values from the MIDAS weather stations at Rothamsted and East Malling. Peaks and troughs of daily soil temperature at 10 cm depth were generally well estimated (Fig. S3a, b). The goodness of fit between observed and simulated daily soil temperature ranged from *R* ^2^ values of 0.73 to 0.82 for Rothamsted and 0.83 to 0.92 for East Malling for different years (Fig. S3c, d). Average air temperature for the potato‐growing season from mid‐April to early October was predicted to increase from the recent period by a mean of 1.9–2.4°C (depending on the location) by the 2040s with the medium‐emission scenario and to 3.9–5.0°C by the 2080s with the high‐emission scenario (Fig. S4a). For the same period, total precipitation decreased by a mean of 8 to 31 mm for the 2040s for the medium‐emission scenario and by 22 to 58 mm for the 2080s for the high‐emission scenario (Fig. S4b). The latter figure represented about 80% of the mean precipitation over the potato‐growing season during the recent years.

**Figure 5:**
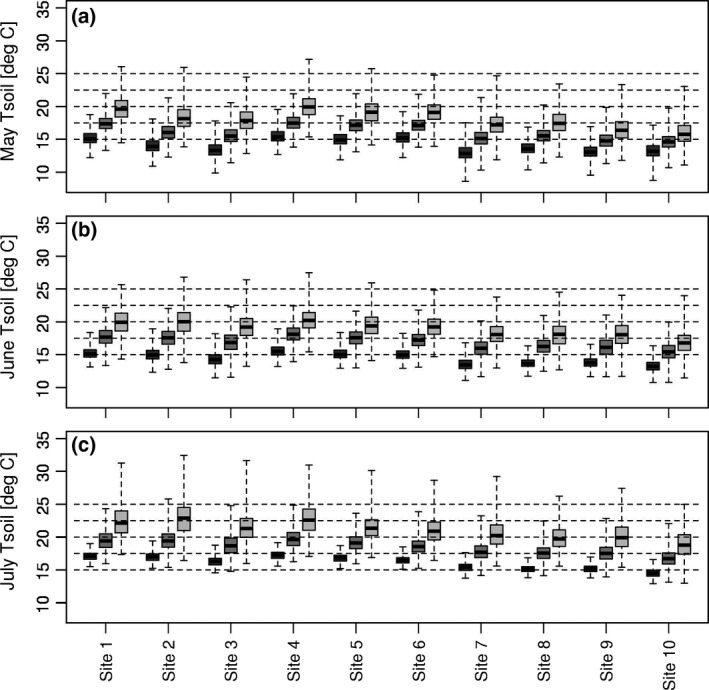
Monthly mean soil temperature (Tsoil) at ten sites as a weighted mean over 10 and 20 cm soil depths for the medium soil type for (a) May, (b) June and (c) July with the high‐emission scenario. Values are for the recent times covering 1961 to 1990 (dark grey), 2040s (medium grey) and 2080s (light grey) and assume a variable canopy and irrigation. The box whisker shows the range for 100 perturbations for each of 50 possible years provided by the weather generator. The whiskers indicate the most extreme values. The horizontal dashed lines indicate the temperatures used in Figure 2 and Fig. S1

Daily soil temperatures were generally higher and more variable at 10 cm than 20 cm depth for both the recent and future simulations (Fig. S5) with a greater effect for the light than the medium soil type (Fig. S6). The medium soil type in combination with variable canopy and irrigation is the most prevalent combination for potato growing in the United Kingdom. With this combination, the mean soil temperature for the medium‐emission scenario increases at 10 cm (20 cm) from the recent to the 2040s by 1.9 to 2.7°C (1.8 to 2.6°C) for June at the ten different locations and 2.0 to 2.6°C (2.0 to 2.6°C) for July (Table S2). The corresponding values at 20 cm soil depth are 1.8 to 2.6°C and 2.0 to 2.6°C. Values for the 2040s and the high‐emissions scenario were similar. For the 2080s, mean increases in soil temperatures might reach 5.1 and 5.0°C for depths of 10 cm and 20 cm, respectively, in June with corresponding values of 5.8 and 5.6°C for July (Table S2). Differences in increases in mean soil temperature varied more depending on the canopy compared to irrigation effects (Table S2). For both June and July, recent median soil temperatures were usually below 15°C for the northern sites and 15°C or slightly above 15°C elsewhere (Figure 5b, c). Even under the high‐emission scenario for the 2040s, the median of the majority of sites in June and all Scottish sites in July remains close to or below 17.5°C. By the 2080s, the median of all sites is above 17.5°C for both the high‐ and medium‐emission scenarios in July (Figure 5c and Fig. S7b) although a difference between them remains.

### 3.5 Estimating the population growth of *Globodera* in response to temperature changes across the United Kingdom

**Figure 6:**
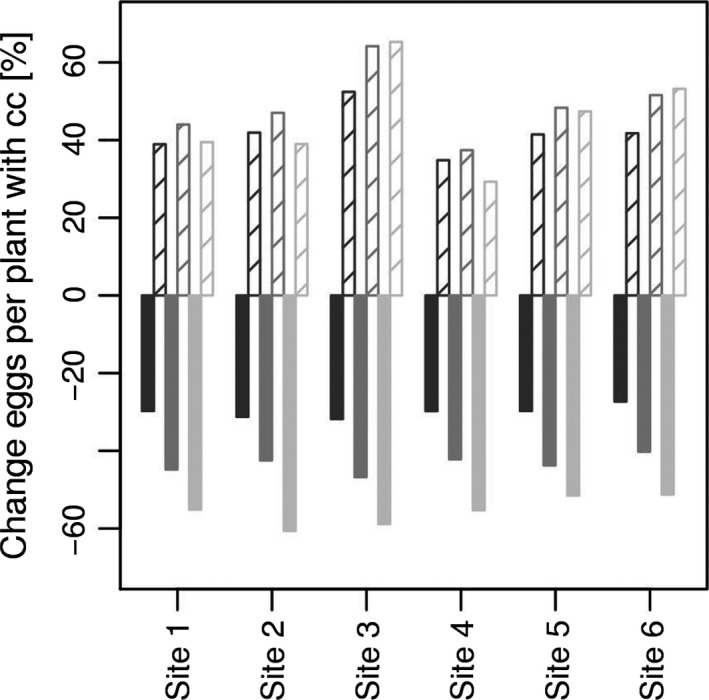
Proportion predicted change for six of the ten sites in the number of eggs per plant using the relationship in Figure 2c and the median values given in Figure 5b, c (mean over June and July) for *Globodera pallida* (filled bar) and *G. rostochiensis* (patterned bar). The change to the 2040s is given in dark grey, to the 2060s in medium grey and to the 2080s in light grey. The remaining four sites have median soil temperature below 15°C in recent times which is below the range studied and are therefore not shown

Figure 6 shows estimates of future *G. pallida* and *G. rostochiensis* population trends across the UK potato‐growing area. It evaluates the median of the recent and future average June and July soil temperatures for the high‐emission scenario (as a weighted mean over 10 and 20 cm depths) and the effects of temperature on the two species (Figure 2). For *G. pallida*, it indicates a percentage reduction in the number of eggs per plant of up to 60% for the six most southern sites. In contrast, increases of 40–70% are predicted for *G. rostochiensis* at the same sites. As the median soil temperature was below 15°C during the recent time period for the four most northern sites, these values need to be verified with experiments covering a broader temperature range than in the current study. Given the estimated relationship of number of eggs per plant in Figure 2c holds, accurate simulation of soil temperatures is crucial as the results differed significantly when a constant canopy was assumed for SoilClim (Fig. S8a, b). Irrigation compared to no irrigation on the other hand did not change the results significantly (Fig. S8c). Interannual variability for mean June and July is high and increases in the future (Figure 5b, c) which indicates that some years will have a larger impact on *Globodera*.

## 4 Discussion

The effect of temperature between 15 and 25°C on female reproductive success differed between *G. pallida* and *G. rostochiensis*. Both number of females per plant and final female size and hence number of eggs per plant were reduced progressively for *G. pallida* at temperatures above 17.5°C (Figure 2 and Fig. S1). In contrast, the number of *G. rostochiensis* females developing on potato was only suppressed above 22.5°C with no decrease in female final size over 15–25°C. This differential effect on number of eggs/g soil produced by the two species was also evident from comparing reproductive success at 15, 22.5 and 25°C on cv Desiree and Maris Peer (Figure 3a). The reduction in reproduction of *G. pallida* was of particular interest in relation to projected future UK summer temperatures and was found to be host independent for seven cultivars studied (Figure 3b).

The reduced number of *G. pallida* females developing may arise from less efficient root invasion, mortality of developing females or a higher proportion of males in unfavourable conditions as sex is determined by environmental conditions in planta (Perry, Wright, & Chitwood, 2013). It seems unlikely to arise from differences in hatch, as there is no substantial effect for either species over the range studied in the recent work by Kaczmarek, Mackenzie, Kettle, and Blok (2014). Furthermore, number of developing females was also reduced at the higher temperature for *G. pallida* when hatched J2s were applied directly to the soil (Figure 2). It could relate to the known differential temperature effects on endogenous lipid reserves used for mobility and root invasion by this nonfeeding stage (Robinson, Atkinson, & Perry, 1987). The small, significant reduction in fecundity must occur after sex determination and suggests suboptimal conditions for the feeding female. Our results for female development are consistent with previous work that suggests that *G. rostochiensis* has a slightly higher thermal optimum (for both number of females and number of eggs per female) compared to *G. pallida* (Berry, Stone, Parrott, & Edwards, 1977). A higher optimum temperature for *G. rostochiensis* has also been found for hatch of its infective juveniles (Foot, 1978; Franco, 1979; Kaczmarek et al., 2014; Robinson et al., 1987). Overall, the results from our study and previous studies establish a preference for *G. pallida* for a lower temperature range compared to *G. rostochiensis*.

The aim of exposing developing females of *Globodera* to diurnal fluctuations from 17.5°C to up to 32.5°C for one week was to examine the likely effect of short periods of high ambient temperatures. Diurnal fluctuations had a significant effect on the development of growing females of *G. pallida* measured one week after this heat stress, but some recovery was evident after a further week. This suggests short periods of high temperature do not suppress multiplication of this species in contrast to sustained high temperatures above about 17.5°C. As previously shown, *G. rostochiensis* has a higher thermal optima compared to *G. pallida* and diurnal fluctuations from 17.5 to 32.5°C had no significant effect on the development of growing females for this species at either time point during recovery.

The SoilClim model simulates the recorded soil temperature accurately at East Malling and Rothamsted for all seven years compared (Fig. S3). This suggests that it provides a useful basis for future projections in conjunction with the weather generator that enabled a spatial resolution of 5 × 5 km. This scale is sufficient for estimating regional effects within the United Kingdom. The projected increases in soil temperature during June and July (Table S2) are in agreement with studies that have investigated past and future trends in soil temperature. Projected increases are at the low end compared to past observed annual soil temperature trends at 30 cm depth in Scotland that reported an increase of 0.30°C per decade (Gregory & Marshall, 2012). Assuming the trend continues into the future, this would equate to an increase of about 1.8 to 2.4°C between the recent time period (1961–1990) and the 2040s. Trends in summer temperature were reported to be higher than trends for annual average soil temperatures for two of three sites in Ireland (García‐Suárez & Butler, 2006). The same was found for some northern forest sites where projected annual mean soil temperatures increased between 1.9 and 3.3°C from the 1971–2000 to the period 2070–99 but increases up to 5.0°C were projected during June (Houle, Bouffard, Duchesne, Logan, & Harvey, 2012).

The increase in frequency of mean soil temperatures above 17.5°C was evident for the six most southern UK sites with both the medium‐ and high‐emission scenarios for July and for June in the latter case. Such temperatures have an adverse effect on the reproductive success of *G. pallida*. The four most northern sites have a lower recent temperature range, and so the increase caused by climate change may not be sufficient to raise soil temperatures to an adverse range for this species. The recent mean soil temperatures in both June and July for all sites are frequently below 17.5°C and are predicted to infrequently exceed 22.5°C under either the medium‐ or high‐emission scenario. The optimal temperature range for *G. rostochiensis* is 17.5–22.5°C suggesting climate change in the United Kingdom will benefit the reproductive success of this species in many years. The SoilClim simulations used for the analysis assume a canopy that has not been affected by nematodes, but some reduction is to be expected by damaging population densities. A less dense canopy would increase temperature fluctuations in summer months that may be sufficient to favour *G. rostochiensis* relative to *G. pallida*.

Combining the data in Figure 2 with the climate change effects suggests a differential effect on the two species. Multiplication of *G. pallida* in the six most southern sites is estimated to be reduced by approximately 30%, 40–50% and 50–60% in 2040s, 2050s and 2080s for the high‐emission scenario (Figure 6). Figure 3a indicates that the reduction in the southern sites might be somewhat lower than presented here, but both data agree on a negative trend. In contrast, similar increases in reproductive success are predicted for *G. rostochiensis* for the same period and conditions but with higher variation between sites. The effect of an increase in mean temperature in the four most northern sites cannot be estimated as current levels were below 15°C and therefore outside the range of the growth experiments. As the medians of future mean soil temperature are between 15 and 20°C, it is anticipated to be insufficient to have either a detrimental effect on *G. pallida* or to favour *G. rostochiensis*. Our results suggest that further work to add a soil temperature parameter to PCN management models (e.g. AHDB Potatoes, http://potatoes.ahdb.org.uk/online-toolbox/pcn-calculator) would improve their utility for anticipating climate change effects for different sites within the United Kingdom. To do this, future work should focus on extending our analysis to distinguish different soil types and to perform tests under field conditions.

Unlike *G. pallida*,* G. rostochiensis* maintained its capacity to multiply at 22.5°C (Figures 2 and 3) and completed a generation in 6–7 weeks postinfection of J2 (Fig. S1). A partial if not full second generation was indicated both by the recovery of more cysts at 16 weeks compared to nine weeks postinfection and by the presence of cysts collected at the second time point with low egg content for their size (Figure 4). Some populations of *G. rostochiensis* in both the United Kingdom (Evans, 1969; Jones, 1950) and Italy (Greco et al., 1988) show a less than complete entry into dormancy of the first generation of eggs and succeed in completing a partial second generation on potato crops. Multiple generations occur for another cyst nematode, *Heterodera schachtii* on the sugar beet crop, which has a more prolonged growing season than potato plants. *Heterodera schachtii* can achieve up to five generations per crop in the warm conditions of the Imperial Valley of California but only typically two generations in the cooler soils that prevail in Northern Europe (Thomason & Fife, 1962). The pest status of *G. rostochiensis* would increase with climate change in the United Kingdom and elsewhere in Europe if a partial second generation became a common response to climate change. This species can be managed by frequent deployment of the qualitative resistance that is present in widely grown cultivars assuming it remains avirulent to that plant defence.

No cultivars with high levels of resistance to *G. pallida* are currently widely grown. Resistant cultivars Innovator and Arsenal are limited to the chipping market in the United Kingdom (http://potatoes.ahdb.org.uk/promotion/chip-skills/Potato-Varieties-Guide). Future control of *G. pallida* would be assisted by cultivars able to withstand future climate change effects in the current growing areas in the south of the United Kingdom. Potato planting may remain at the same time of year because of other husbandry constraints (Brown, Towers, Rivington, & Black, 2008; Gregory & Marshall, 2012). If a shift in planting potatoes towards earlier dates occurs in the future, this would place the start of female development into May. The soil temperatures during May would favour *G. pallida* as they are cooler than in June and July with the median of the mean monthly soil temperature at or below 17.5°C until the 2040s for all sites (Figure 5a). Potato yields in England are predicted to increase from approximately 2.9 to 6.5% by mid‐century due to warmer temperatures, assuming current nitrogen management and unconstrained water availability. Current irrigation schemes will not meet needs to achieve future yields in approximately 50% of years with 14 to 30% more water required by mid‐century (Daccache et al., 2011). The importance of PCN will be increased if the crop experiences water stress more often as the parasite reduces water acquisition by the root system (Fatemy & Evans, 1986). A shift to the north and west would lessen irrigation demands (Downing et al., 2003), but the effect may be slow because of the investment levels required of successful potato growers (Daccache et al., 2012).

It is generally assumed that PCN was introduced from S. America to Europe in about 1850. Consequently, with a single generation per potato crop and a common rotation of 3–7 years in the United Kingdom (http://potatoes.ahdb.org.uk/sites/default/files/publication_upload/pcnOfficialControlProgramme.pdf), there have only been about 25 to 60 generations in the country for the founding populations. The limited number of generations may explain the continued optimal temperature difference of the two species as defined during their co‐evolution with Solanaceae in S. America over 15–21 × 10^6^ years. Some animals are likely to overcome the impact of climate change by range changes (Hof, Levinsky, AraÚJo, & Rahbek, 2011), but this does not apply to *G. pallida* because it is already present throughout much of the United Kingdom (Minnis et al., 2002). The prevailing consensus is that climate change normally outpaces microevolution processes that enable the adaptation required to remain at some localities (Hof et al., 2011). Exceptions include *Daphnia magna* which has a rapid life cycle and lives in shallow pools susceptible to changes in water temperature. The planktonic crustacean showed a 2°C increase in the maximum temperature at which it shows locomotor activity over a two‐year period (Geerts et al., 2015). It seems unlikely that *Globodera* will have a similar capacity to achieve such rapid microevolution given its infrequent reproduction.

Our work suggests dual priorities for potato plant breeders, that is to exploit the thermal limits of *G. pallida* and continued incorporation of resistance against *G. rostochiensis* to counter possible benefits to it from warmer temperatures in the United Kingdom.
