# Supplementary material for: Climate change is predicted to alter the current pest status of Globodera pallida and G. rostochiensis in the United Kingdom
Source: Glob Chang Biol. 2017 Mar 30;23(11):4497–507. doi: 10.1111/gcb.13676 (PMC5655772; doi:10.1111/gcb.13676)
Supplement: Supplementary file 1 [file GCB-23-4497-s001.docx]

**Supplementary information**

**Climate change is predicted to alter the current pest status of *Globodera pallida* and *G. rostochiensis* in the UK**

Laura M. Jones, Ann-Kristin Koehler, Mirek Trnka, Jan Balek, Andrew J. Challinor, Howard J. Atkinson, Peter E. Urwin

**Table S1:** Selected UK 5x5 km grid cells for the analysis (according to UKCP weather generator latitude and longitude) (Fig. 1). Verification if the selected grid cells lie in agricultural areas and their altitude was determined using [www.gridreferencefinder.com](http://www.gridreferencefinder.com).

| **Site Name** | **Location / county** | **Latitude** | **Longitude** | **Altitude (m)** |
| --- | --- | --- | --- | --- |
| Site 1 | Suffolk | 52.2463 | 1.5408 | 23 |
| Site 2 | Herefordshire | 52.2547 | -2.9169 | 150 |
| Site 3 | Shropshire | 52.5697 | -2.7026 | 243 |
| Site 4 | Lincolnshire | 52.8260 | 0.1044 | 4 |
| Site 5 | Norfolk | 52.8422 | 1.3747 | 30 |
| Site 6 | Lancashire | 53.6479 | -2.9388 | 4 |
| Site 7 | North Yorkshire | 54.0513 | -0.7251 | 190 |
| Site 8 | East Lothian | 56.0322 | -2.7466 | 48 |
| Site 9 | Angus | 56.7557 | -2.7686 | 86 |
| Site 10 | Aberdeenshire | 57.5600 | -2.3840 | 133 |

**Table S2:** Influence of management on increases in mean soil temperatures at 10 and 20 cm depths between the recent and the future time periods for the medium soil type for June (July). Given are minimum and maximum increases which depend on the location. irr = irrigation.

|  | **Medium emissions scenario** | | | | **High emissions scenario** | | | |
| --- | --- | --- | --- | --- | --- | --- | --- | --- |
|  | **2040s** | | **2080s** | | **2040s** | | **2080s** | |
|  | **10cm** | **20cm** | **10cm** | **20cm** | **10cm** | **20cm** | **10cm** | **20cm** |
| **constant canopy, no irr** | 1.7 to 2.7 (2.2 to 3.0) | 1.7 to 2.5 (2.1 to 2.9) | 3.2 to 4.5 (3.8 to 5.0) | 2.9 to 4.3 (3.7 to 4.9) | 2.1 to 2.8 (2.4 to 2.9) | 2.0 to 2.6 (2.3 to 2.8) | 3.6 to 5.4 (4.8 to 6.6) | 3.4 to 5.1 (4.6 to 6.4) |
| **constant canopy, irr** | 1.7 to 2.6 (2.1 to 2.9) | 1.6 to 2.4 (2.0 to 2.9) | 3.1 to 4.4 (3.7 to 4.9) | 2.9 to 4.2 (3.5 to 4.8) | 2.0 to 2.7 (2.3 to 2.8) | 1.9 to 2.5 (2.3 to 2.7) | 3.5 to 5.2 (4.6 to 6.4) | 3.3 to 5.0 (4.4 to 6.2) |
| **variable canopy, no irr** | 1.9 to 2.7 (2.0 to 2.6) | 1.8 to 2.6 (2.0 to 2.6) | 3.3 to 4.3 (3.5 to 4.4) | 3.1 to 4.2 (3.5 to 4.3) | 2.2 to 2.7 (2.1 to 2.5) | 2.1 to 2.6 (2.1 to 2.5) | 3.7 to 5.1 (4.4 to 5.8) | 3.5 to 5.0 (4.3 to 5.6) |
| **variable canopy, irr** | 1.9 to 2.7 (2.0 to 2.7) | 1.8 to 2.6 (2.0 to 2.6) | 3.3 to 4.3 (3.5 to 4.4) | 3.2 to 4.2 (3.5 to 4.4) | 2.2 to 2.7 (2.1 to 2.5) | 2.1 to 2.6 (2.1 to 2.5) | 3.7 to 5.1 (4.5 to 5.9) | 3.6 to 5.0 (4.3 to 5.7) |

**
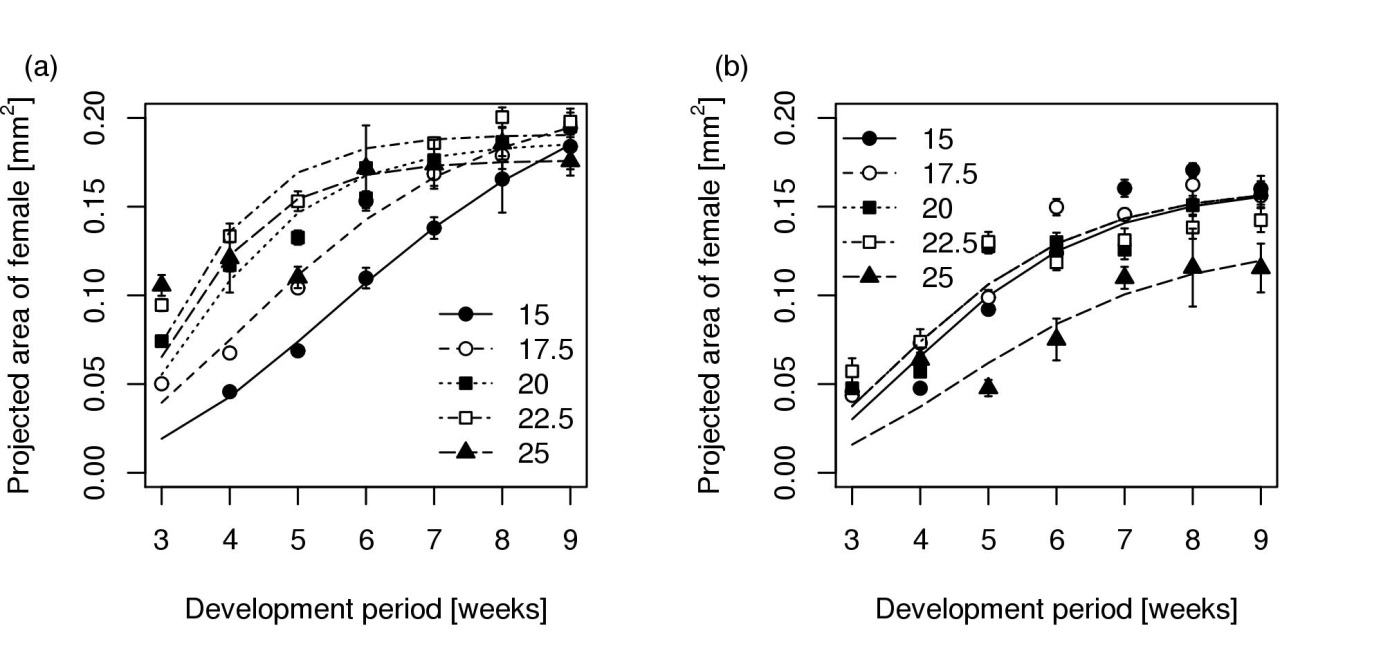
**

**Figure S1:** (a) Changes in projected surface area of female *G. rostochiensis* developing at 15 to 25 °C measured in weeks after addition of infective juveniles to soil. Areas were measured from when females became abundant on the root surface until growth was completed. (b) as (a) but for *G. pallida*.

**
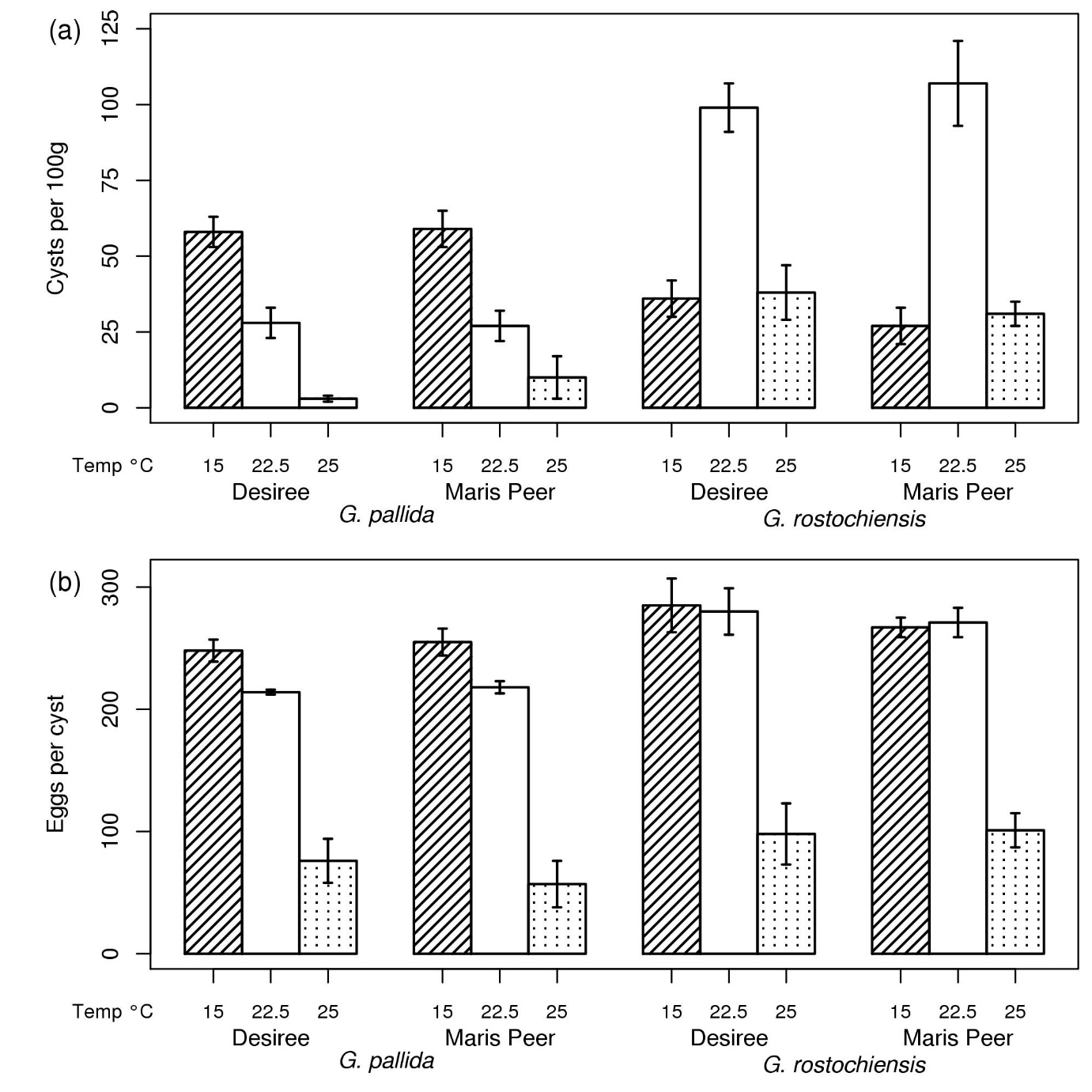
**

**Figure S2:** Cysts per 100 g soil (a) and eggs per cyst (b) of *G. pallida* and *G. rostochiensis* at 15, 22.5 and 25 °C for cultivars Desiree and Maris peer. Values are means ± SEM.


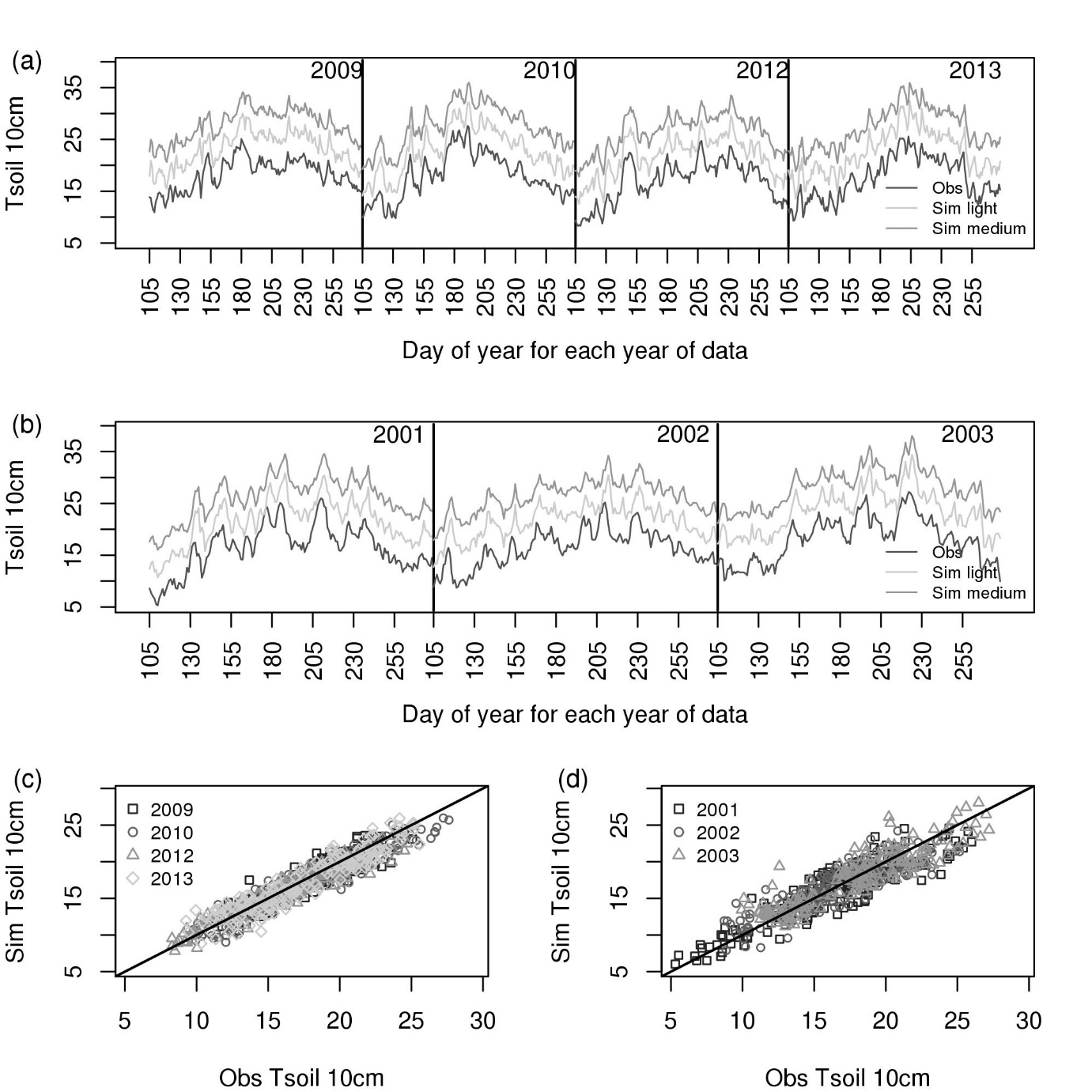


**Figure S3:** Observed (Obs) and simulated (Sim) soil temperature (Tsoil) in 10 cm depth for two UK MIDAS weather stations for years with good quality data. (a) and (b) show the time series during the UK potato growing season (mid April to early October) comparing the observed data with light and medium soil type simulations. The light (medium) soil type simulations have +5 °C (+10 °C) added to better compare peaks and troughs. The black vertical lines delineate different years. (c) and (d) show the observed versus the simulated soil temperature for the same years as shown in (a) and (b) respectively for the medium soil type. The black line is the 1:1 line.


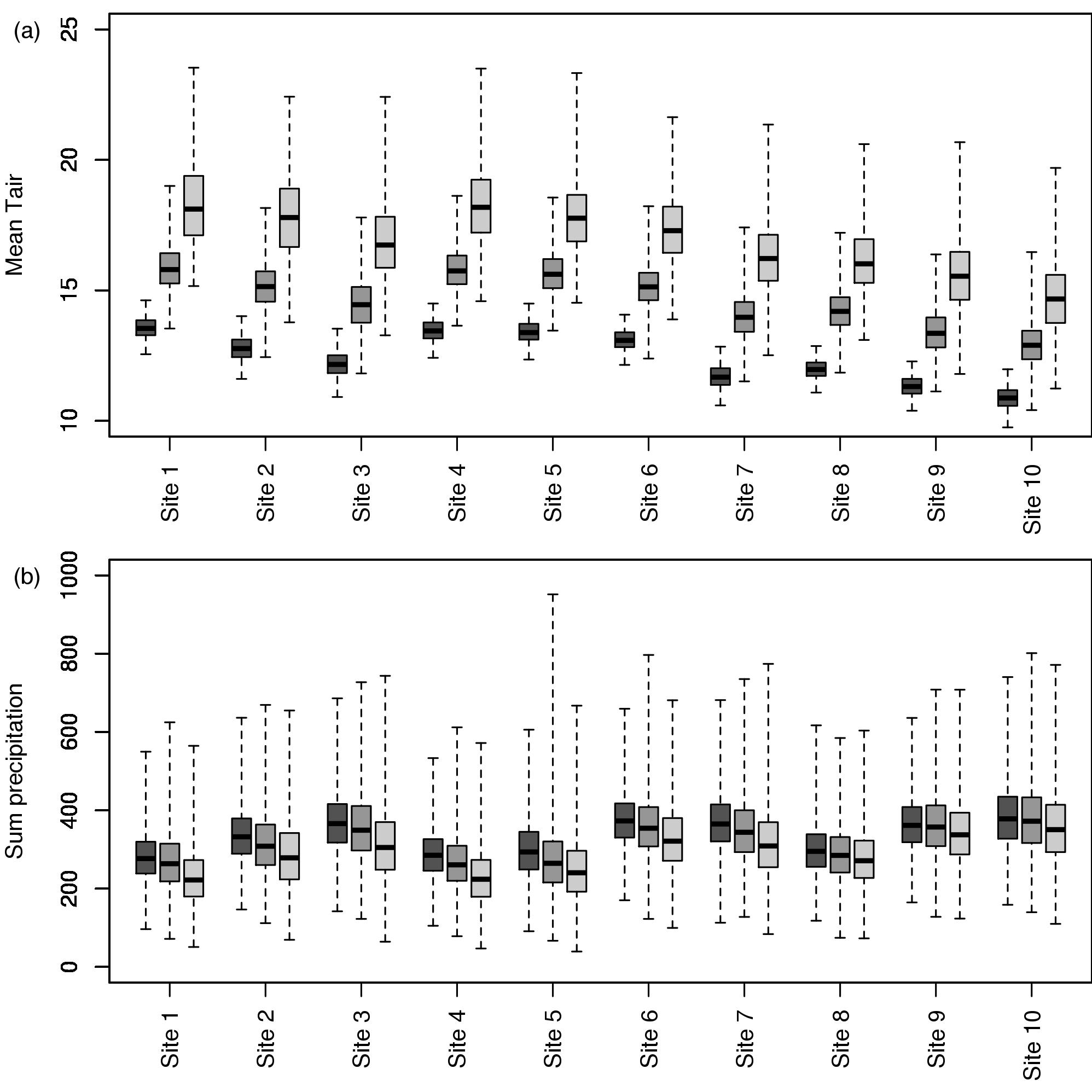


**Figure S4:** Mean air temperature (Tair) (a) and the sum of precipitation (b) at the ten sites with the high emissions scenario for the potato growing season from mid April to early October. Values are for the recent times covering 1961 to 1990 (dark grey), 2040s (medium grey), and 2080s (light grey). The box-whisker shows the range for 100 perturbations for each of 50 possible years provided by the weather generator. The whiskers indicate most extreme values.

**
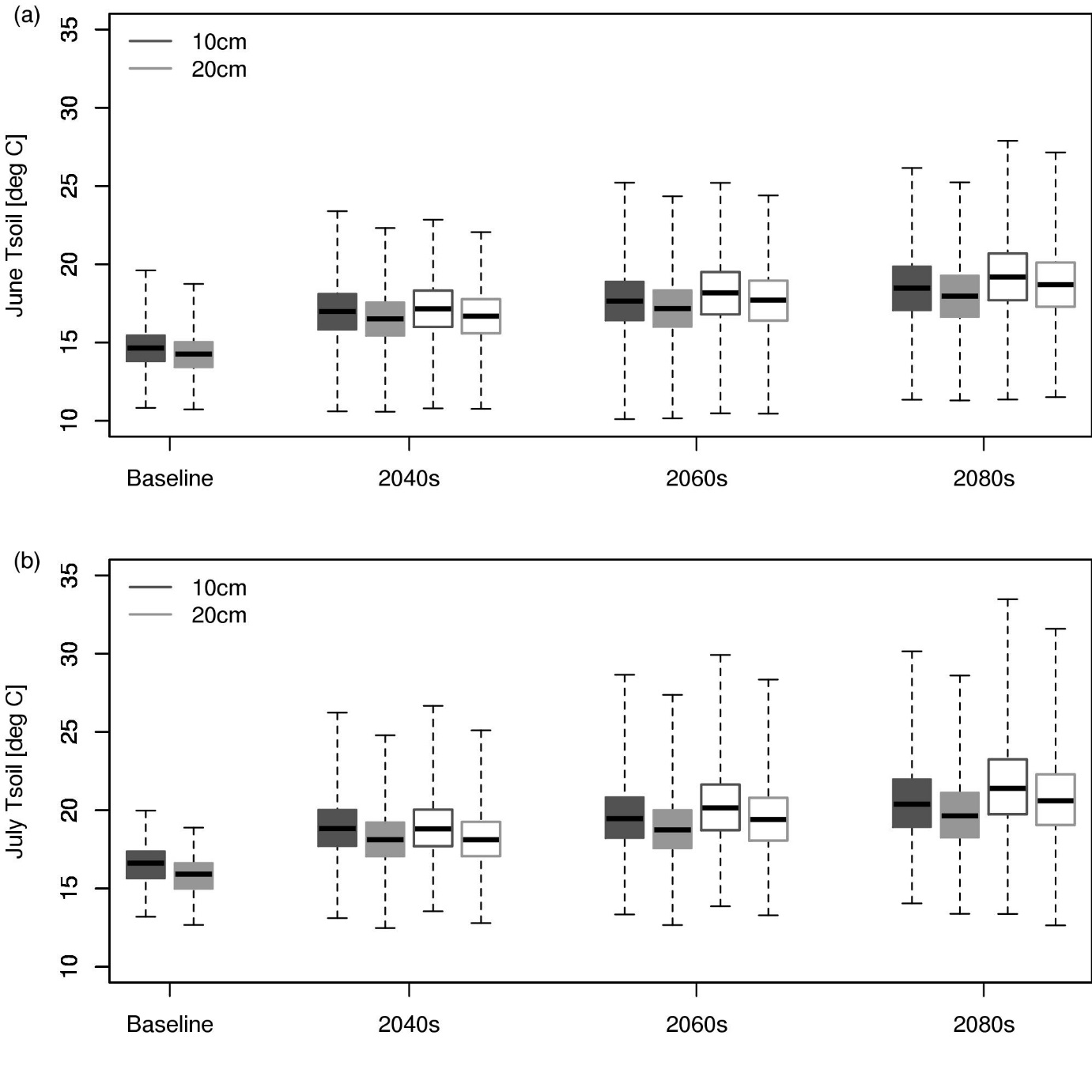
**

**Figure S5:** Monthly mean soil temperature (Tsoil) in 10 and 20 cm depth for the medium soil type for (a) June and (b) July. Values are for the recent times covering 1961 to 1990 and the 2040s, 2060s, and 2080s and assume a variable canopy and irrigation. The filled boxes are for the mid emissions scenario whereas the open boxes are for the high emissions scenario. The box-whisker shows the range for the ten selected sites combined with 100 perturbations for each of 50 possible years provided by the weather generator. The whiskers indicate the most extreme values.


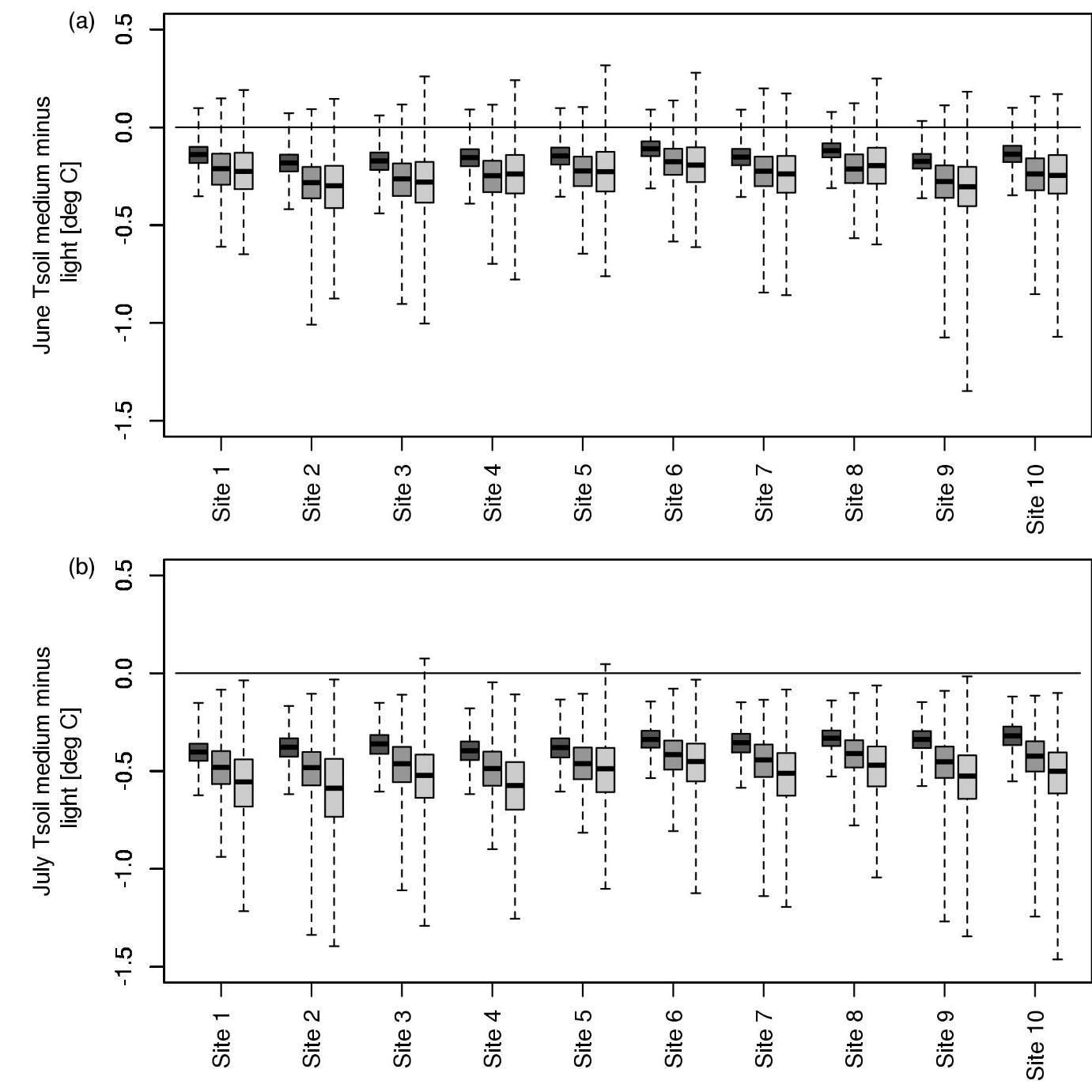


**Figure S6:** The difference between the medium and the light soil type for monthly mean soil temperature (Tsoil) at the ten sites as a weighted average over 10 and 20 cm soil depths for (a) June and (b) July with the high emissions scenario. Values are for the recent times 1961 to 1990 (dark grey), 2040s (medium grey), and 2080s (light grey) and assume a variable canopy and irrigation. The box-whisker shows the range for 100 perturbations for each of 50 possible years provided by the weather generator. The whiskers indicate most extreme values.

**
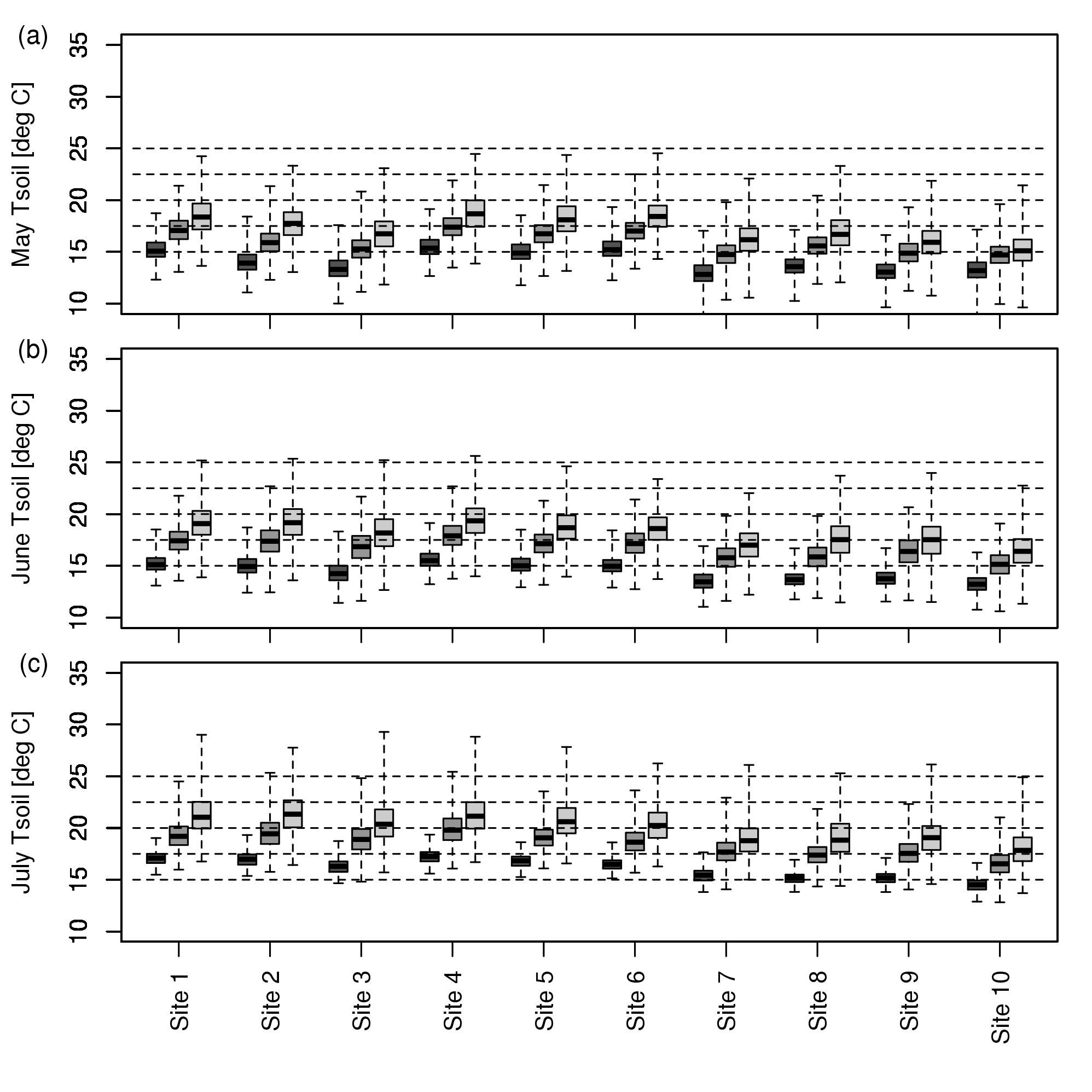
**

**Figure S7:** As Fig. 6 but for the medium instead of the high emissions scenario.

**
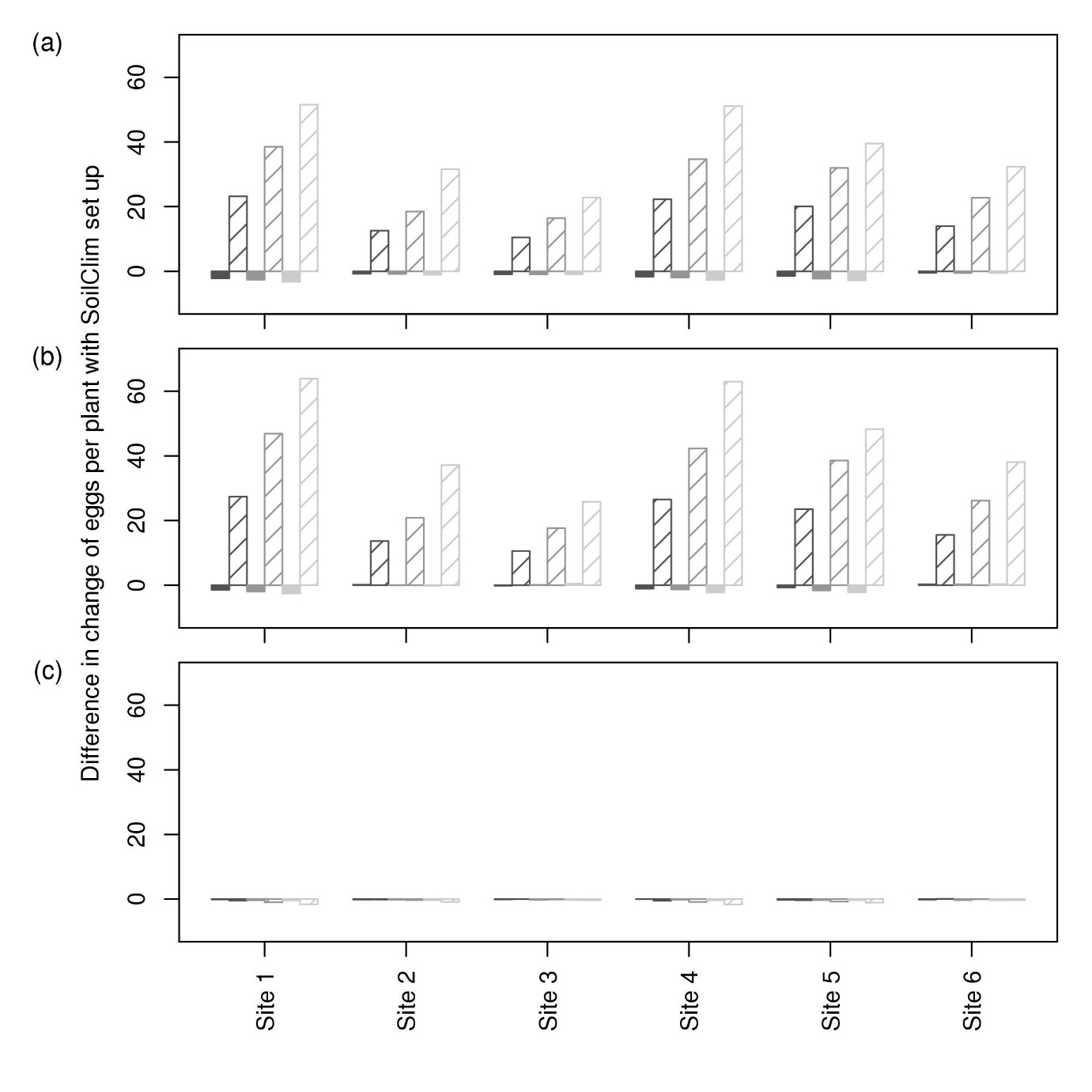
**

**Figure S8:** Differences in predicted eggs per plant [%] for *G. pallida* and *G. rostochiensis* using four management set-ups for the soil temperature simulations. Variable canopy with irrigation is assumed to be the default. Calculations are done as in Fig. 7 using the median values over June and July and the conditions given in Fig. 6. (a) shows constant canopy with irrigation, (b) constant canopy without irrigation, and (c) variable canopy without irrigation. Data are shown for *G. pallida* (filled bar) and *G. rostochiensis* (patterned bar) for the 2040s (dark grey), 2060s (mid grey), and 2080s (light grey).
